# Manipulating the plant mycobiome to enhance resilience: Ecological and evolutionary opportunities and challenges

**DOI:** 10.1371/journal.ppat.1011816

**Published:** 2023-12-14

**Authors:** Christine V. Hawkes, Xavious Allen, Peter Balint-Kurti, Christina Cowger

**Affiliations:** 1 Department of Plant and Microbial Biology, North Carolina State University, Raleigh, North Carolina, United States of America; 2 Plant Science Research Unit, USDA-ARS, Raleigh, North Carolina, United States of America; 3 Department of Entomology and Plant Pathology, North Carolina State University, Raleigh, North Carolina, United States of America; University of Maryland, Baltimore, UNITED STATES

## Introduction

Recent evidence supports a substantial role for fungal symbionts in mediating plant stress phenotypes, including plant drought tolerance and defense against pathogens [1,2], which has generated interest in manipulating these fungi to enhance plant resilience. Methods to manipulate the plant mycobiome can (1) leverage existing fungal communities by promoting beneficial taxa or functions, or (2) disrupt existing fungal communities by introducing novel (or genetically modified) taxa. Manipulation of existing communities has a long history in agriculture, with, for example, crop rotations to help suppress soil-borne fungal disease [3]. More recently, entire foliar fungal communities from healthy plants were successfully transplanted to reduce rust infections in a critically endangered tree [4]. In the near future, applications that target chemical signaling to stimulate plant colonization by beneficial fungi or to induce their benefits for the plant may become more widespread [5]. In the longer term, plant breeding or genetic manipulation to produce varieties that support more beneficial mycobiomes may be an effective strategy [6], given that historically, breeding has inadvertently shifted plant-fungal associations [7].

Most current mycobiome interventions (particularly in the agricultural bioproduct market) introduce locally novel fungi. While many of these inoculants demonstrate benefits in controlled conditions, success in the field can be limited. For example, in plant restoration experiments and in tests with crops, commercial mycorrhizal fungal inocula rarely improve plant establishment or growth [8,9]. Moreover, introducing novel taxa can result in detrimental impacts to local plants and ecosystems, with effects such as invasion of the surrounding landscape by ectomycorrhizal fungi and pine trees originally introduced for plantation forestry [10].

**Fig 1:**
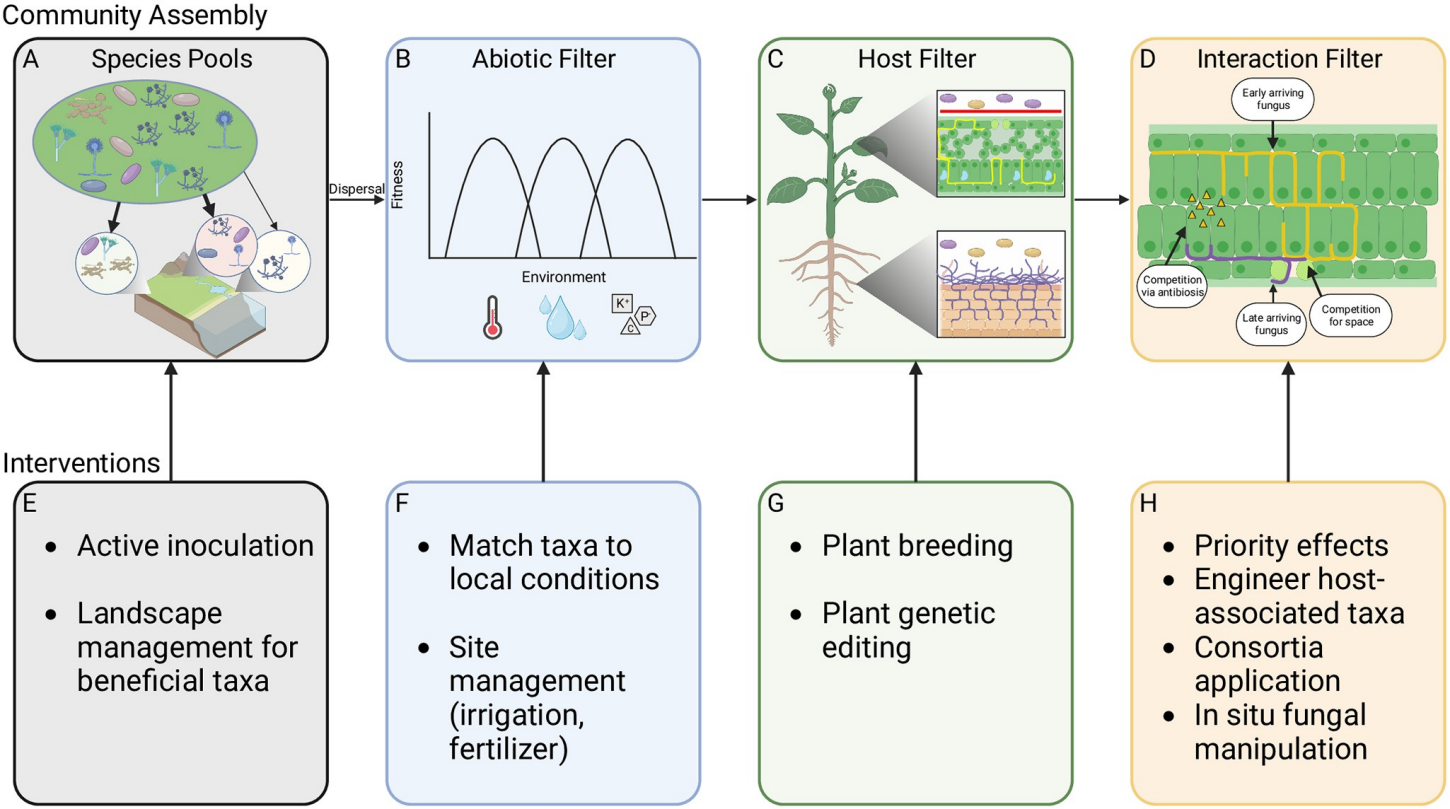
Mycobiome assembly processes and intervention strategies. Fungi are (**A**) dispersed from local and regional species pools to local sites, where they are filtered (**B**) by abiotic (environmental) characteristics such as temperature, soil moisture, and soil nutrient content, (**C**) by host characteristics, and (**D**) by interactions with other microorganisms in and on the host. Intervention strategies (**E**-**H**) can be applied at each step to influence the action of each filter. (**A**) Species pools can be modified through (**E**) direct inoculation of beneficial taxa or landscape management to promote beneficial taxa. (**B**) Abiotic filtering can be modified through (**F**) introduction of taxa successful in local conditions as well as management of the site to promote specific taxa. (**C**) Modification of host traits or genetics using (**G**) conventional or biotechnological approaches can promote or exclude fungal taxa. (**H**) Tailoring which fungi interact and when is the goal of interventions to the (**D**) interaction filter: Priority effects can be exploited to give target fungi a “head start,” consortia comprised of coexisting fungi with complementary benefits may be applied, and engineering of host-associated taxa in the lab or in situ may be used to adjust fungal interactions. *Created with* Biorender.com.

To manipulate mycobiomes effectively and safely in ways that improve plant stress tolerance and resilience in real-world ecosystems requires a broad understanding of these plant-fungal interactions, ranging from their ecology and evolution to the molecular mechanisms underlying observed phenotypes. Here, we address the major hurdles and opportunities for mycobiome management (Fig 1), while noting that evidence for success of any one strategy remains limited.

## Manipulating community assembly processes

The plant mycobiome results from fungi that arrive via dispersal, pass through environmental and host filters, and survive interactions with the preexisting microbial taxa (Fig 1A–1D). Each of these steps in the assembly process represents an opportunity for mycobiome manipulation (Fig 1E–1H). The historical paradigm of “everything is everywhere” has been replaced with an understanding that some fungi are widespread, while distributions of others are limited by low rates of dispersal (Fig 1A) [11]. Thus, tipping local species pools toward preferred taxa by active inoculation or by selection via environmental modification may engineer the mycobiome to some degree, particularly in degraded landscapes with limited local fungal diversity (Fig 1E). Attempts to generate disease-suppressive soils in agriculture are a long-standing example of manipulating local species pools to limit pathogen availability for plant colonization [12]. In modern biocontrol, inoculations with the generalist fungus *Trichoderma* are common and often successful, likely due to its diverse lifestyles [13]. Environmental modifications might take the form of planned mosaics of agricultural and natural vegetation to generate more diverse source pools of fungi, as seen for foliar fungi in crops with more abundant natural vegetation located within 1 km of the farm [14].

Fungal abiotic tolerances determine how they respond to environmental filters (Fig 1B), meaning that best practices for mycobiome manipulation should match fungal tolerances or requirements to local conditions (Fig 1F). Host filters (Fig 1C), in contrast, depend on plant ecological and genetic traits [15,16]. As noted above, host filters could be modified by targeting mechanisms of plant-fungal interactions through breeding or gene editing to either increase or decrease filter stringency (Fig 1G). This might mean altering plant traits such as leaf thickness, leaf nutrient content, the composition of leaf waxes [15], or the quantity or composition of exudates [17]. Alternatively, host filters may be altered by altering recognition and signaling pathways [18]. However, care must be taken with such approaches given that beneficial and pathogenic taxa can rely on the same interaction pathways [19].

Finally, establishing novel taxa in existing communities is challenging due to competitive and antagonistic interactions with taxa that already occupy the plant (Fig 1D). To limit the influence of negative interactions, approaches such as seed inoculation or application to newly emerged leaves provide an opportunity for target fungi to establish prior to arrival of fungi from the surrounding environment (Fig 1H). However, these “priority effects” may be transient if pioneer fungi cannot compete with later-arriving taxa [20]. An alternative is to use extant fungal taxa isolated from the host to engineer synthetic consortia, which then have the advantage of being able to colonize and coexist on the plant [21].

## Host defense

Host defenses operate as additional host filters (Fig 1C) but are active rather than passive gatekeepers, allowing beneficial fungi to enter plants while attempting to exclude unwanted pathogens. In general, the salicylic acid pathway is effective against biotrophic fungi, while the jasmonic acid pathway is effective against necrotrophic fungi [22]. Mycorrhizal and endophytic fungi are able to avoid or suppress host defense systems to establish symbiotic relationships. Although we do not fully understand this process, it appears to rely on specific signaling between plant and fungus that results in a mutualistic rather than immune response by the host [18].

There is extensive evidence that beneficial fungal symbionts can alter disease severity through multiple mechanisms ranging from direct competition with the pathogen to enhanced host resource availability for defense [23,24]. Of particular interest is that symbiotic fungal colonization can result in priming, or stronger induction of host defense responses, upon an appropriate subsequent stimulus [25–27].

Manipulating the mycobiome to enhance priming may be one way to limit disease. For example, inoculation with the well-known endophyte *Trichoderma* can enhance priming over days to weeks, with strain-specific regulation of systemic defense genes related to abscisic acid, jasmonic acid, and salicylic acid metabolism, among others [26]. Using inoculation with beneficial fungi as a priming tool to protect plants from disease may be cost-effective, but perhaps only when there is disease pressure, given expected trade-offs in allocation to growth versus defense [28]. Beyond using the approaches outlined above to alter community assembly to favor such beneficial taxa, efforts are underway to use CRISPR/Cas9 editing of both plant and fungal genomes to enhance host resistance [29].

## Maintenance of beneficial mycobiomes

In some cases, mycobiome manipulation may be intended to be transient, perhaps for addressing temporary stressors in crop management. In other cases, the aim may be to initiate persistent relationships, as might occur when preparing the host plant for shifting climate conditions. Once the mycobiome is established, how it interacts with the host can determine the short- and long-term trajectory of the fungal community. Any fungal symbiont that improves the fitness of its host should be able to spread and persist in the plant population, but evolutionary mechanisms must come into play to promote beneficial versus harmful relationships over time [30]. One classic mechanism that can stabilize mutualisms is host preferential allocation (or withholding) of resources to more (or less) beneficial partners—known as partner choice or partner discrimination. A well-known example is the trade-based relationship, such as in arbuscular mycorrhizal fungi, where preferential plant photosynthate allocation to more beneficial fungi can facilitate some taxa at the expense of others [31]. The coarse scale of allocation, however, means that mutualisms are likely maintained as a component of nonmutualist consortia at the community level [32], challenging efforts to precisely manipulate allocation pathways over the medium and long term. Recent work suggests that plant-fungal trade goes beyond the traditional mycorrhizas to other co-occurring root fungi [33,34], further supporting the idea that the scale of host allocation will play a key role in the longer-term stabilization of mutualism. One challenge will be to understand the potential evolutionary mechanisms that might support nonnutritional plant-fungal relationships, particularly when fungi are not obligate symbionts and have life stages outside the host.

## Functional consistency

Beyond assembling and maintaining target mycobiomes, predictable function is needed to consistently achieve desired host benefits. Yet, fungi are notoriously plastic in their behavior, which depends on factors such as host and environment. For example, pathogenic fungi found in many dicots can act as beneficial endophytes in some grass crops [35], and endophytic fungi that enhance grass survival under drought can be parasitic in well-watered conditions [36]. Other fungi are locally adapted such that function degrades away from the “home” environment, as found for some arbuscular mycorrhizal fungi adapted to local edaphic conditions [37] and *Neurospora* populations adapted to local temperatures [38]. We may be able to take advantage of fungal adaptation by using direct inoculations to address specific needs in certain conditions (Fig 1F). However, in many cases, local adaptation will be a barrier to broad application and raises the question of whether or not we can engineer strains or consortia for generating precise effects in a wide range of environments. Fungal domestication through artificial selection has been successful for many industrial microbes experiencing a restricted set of conditions [39], but the same approaches are unlikely to be effective for fungi that live in host-associated, complex communities under fluctuating environmental conditions where they are unlikely to consistently be the fittest strains. More promising are new methods that allow for engineering native microbial communities in situ (Fig 1H) to achieve specific functions, relying on, for example, horizontal gene transfer via mobile genetic elements or phage [21]. In situ approaches could support transient adjustments on an as-needed basis to deal with, for example, short-term drought. Currently, in situ manipulations have only been tested in bacteria and additional work would be needed to enable similar strategies for the plant mycobiome.

## Conclusions

Plant mycobiome manipulation is a promising tool for enhancing plant resilience in a more stressful future. The most successful approaches are likely to be those that combine molecular and genetic tools with an understanding of the ecology and evolution of plant-fungal interactions.
